# Head-to-Head Comparison between [^68^Ga]Ga-DOTA.SA.FAPi and [^18^F]F-FDG PET/CT Imaging in Patients with Breast Cancer

**DOI:** 10.3390/ph16040521

**Published:** 2023-03-31

**Authors:** Sanjana Ballal, Madhav P. Yadav, Frank Roesch, Nicky Wakade, Shobhana Raju, Parvind Sheokand, Prashant Mishra, Euy Sung Moon, Madhavi Tripathi, Marcel Martin, Chandrasekhar Bal

**Affiliations:** 1 Department of Nuclear Medicine, All India Institute of Medical Sciences, New Delhi 110029, India; mail.sanjanaballal87@gmail.com (S.B.);; 2 Department of Chemistry—TRIGA Site, Johannes Gutenberg University, 55099 Mainz, Germany

**Keywords:** [^68^Ga]Ga-DOTA.SA.FAPi, [^18^F]F-FDG PET/CT, breast cancer

## Abstract

This study aimed to compare the diagnostic performance of [^68^Ga]Ga-DOTA.SA.FAPi with that of [^18^F]F-FDG PET/CT in detecting primary and metastatic lesions of breast cancer. [^18^F]F-FDG and [^68^Ga]Ga-DOTA.SA.FAPi PET/CT scans of histologically proven breast cancer patients were compared according to patient-based and lesion-based analysis. Forty-seven patients with a mean age of 44.8 ± 9.9 years (range: 31–66 years) were evaluated. A total of 85% of patients had invasive ductal carcinoma, and 15% had invasive lobular carcinoma. The tracer uptake [SULpeak, SULavg, and the median tumor-to-background ratio (TBR)] was significantly higher in [^68^Ga]Ga-DOTA.SA.FAPi than with [^18^F]F-FDG PET/CT for lymph nodes, pleural metastases, and liver lesions (*p* < 0.05). However, for brain metastasis, only the median TBR was significantly higher (*p* < 0.05) compared to [^18^F]F-FDG. In patient-based analysis the sensitivity of [^68^Ga]Ga-DOTA.SA.FAPi PET/CT was higher, but not significant than that of [^18^F]F-FDG PET/CT in the detection of both primary tumors and metastatic lesions. According to lesion-based analysis, on diagnostic CT, 47 patients had 44 primary tumors, 248 lymph nodes, 15 pleural, 88 liver, and 42 brain metastases. [^68^Ga]Ga-DOTA.SA.FAPi scan identified more abnormal lesions than [^18^F]F-FDG in all the primary and metastatic sites with a maximum marked difference in the primary site [88.6% vs. 81.8%; *p*-0.001], lymph nodes [89.1% vs. 83.8%; *p*-0.0001], pleural metastases [93.3% vs. 73%; *p*-0.096] and brain metastasis [100% vs. 59.5%; *p*-0.0001]. [^68^Ga]Ga-DOTA.SA.FAPi PET/CT was superior to [^18^F]F-FDG PET/CT in the imaging of breast cancers.

## 1. Introduction

Breast cancer is the most common malignancy in women. With the advent of several advanced imaging and treatment options, death due to breast cancer has declined over time [1]. Among the imaging modalities, [^18^F]F-FDG PET/CT is the most widely used systemic option for the diagnosis, staging, response assessment, and prognostication of breast cancers. As compared to other conventional imaging modalities, the sensitivity of [^18^F]F-FDG is remarkable with an estimated sensitivity of 97%. However, it exhibits a rather low specificity of 77% in breast cancer patients due to the high false positive results of uptake in inflammatory lymph nodes, and specifically low detection rates in micrometastases and sclerotic healed bone lesions [2].

Hence, there is an unmet need to evaluate new molecular whole-body imaging techniques based on cell-specific oncological targets from a theranostic point of view in end-stage breast cancer patients. Since 2018, imaging fibroblast activation protein (FAP) expression in tumor microenvironment utilizing fibroblast activation protein inhibitors (FAPi) labeled with gallium-68 has gained interest and an increasing number of studies have attempted to explore this modality in various cancers.

Huang et al. [3] observed FAP expression in breast cancer but not in normal breast tissue and proved that significant FAP expression was paralleled by increased tumor growth rates in a mouse model of human breast cancer. Their further investigations lead to significant findings that the proteolytic activity of FAP participates in matrix degradation, but other functions of the protein stimulate increased tumor growth.

One of the first investigations found [^68^Ga]Ga-FAPI-04 PET/CT useful in the detection of breast cancers [4]. Another retrospective study involving 48 breast cancer patients found that [^68^Ga]Ga-FAPI-04 PET/CT imaging revealed more lesions in all classified regions and had higher uptake values than [^18^F]F-FDG PET/CT and reported a remarkable improvement in the sensitivity and specificity compared to [^18^F]F-FDG [5]. At our institute, we use the squaric acid-based FAP inhibitor DOTA.SA.FAPi, which additionally has a theranostic option of treatment with DOTAGA. (SA.FAPi)_2_ dimer [6,7,8,9,10,11,12]. However, not much has been reported on this molecule in breast cancer. Accordingly, in this study, we compare the diagnostic efficacy of [^68^Ga]Ga-DOTA.SA.FAPi PET/CT with [^18^F]F-FDG PET/CT in breast cancer patients.

## 2. Results

### 2.1. Patients

A total of 47 breast cancer patients were included in the study and underwent [^18^F]F-FDG and [^68^Ga]Ga-DOTA.SA.FAPi PET/CT scans. The mean age of the patients was 44.8 ± 9.9 years (range: 31–66 years). A total of 85% had invasive ductal carcinoma, and 15% had invasive lobular carcinoma. Twelve patients had the triple-negative disease, 17 had HER2-positive, and 18 had HER2-negative disease. A total of 95.5% of patients had distant metastases, and in three patients the disease was locally advanced. Forty patients underwent surgery and the remaining did not. The majority (80.8%, *n* = 38) of the patients underwent chemotherapy and five among them underwent two lines of chemotherapy. Local RT, hormonal treatment, immunotherapy, tyrosine kinase inhibitors, and palliative RT for distant metastases were administered to 23.6%, 17.0%, 12.7%, 10.6%, and 8.5% of patients, respectively. Scans were conducted in 39 patients for re-staging after anti-cancer treatments and in eight patients for initial staging/workup.

### 2.2. Patient-Based Detection Rate Analysis

The primary tumor detection rates for [^68^Ga]Ga-DOTA.SA.FAPi and [^18^F]F-FDG PET/CT were similar in patient-based analyses (96.6% [29/30] vs. 90.0% [27/30], *p*-0.3107), as were the rates of detection for lymph node (97.5% [40/41] vs. 92.6% [38/41], *p*-0.309), pulmonary (96.1% [25/26] vs. 96.1% [25/26], *p*-1.000), pleural (100% [8/8] vs. 75% [6/8], 0.1432), hepatic (100% [17/17] vs. 100% [17/17], *p*-1.000), and bone metastases (100% [25/25] vs. 96% [24/25], *p*-0.865). Diffuse non-specific [^18^F]F-FDG uptake in the entire skeleton was noted in two patients. A total of 2.6% and 10.9% of patients showed a prevalence of mixed uptake pattern (TP + FP + TN + FN) in the lung nodules on [^68^Ga]Ga-DOTA.SA.FAPi and [^18^F]F-FDG PET/CT scans respectively (Table 1).

### 2.3. Lesion-Based Analysis

In forty-seven patients, 44 primary tumors, 248 lymph nodes, 15 pleural, 88 liver, and 42 brain metastases were identified on diagnostic CT. [^68^Ga]Ga-DOTA.SA.FAPi scan identified more abnormal lesions than [^18^F]F-FDG in all the primary and metastatic sites with a maximum marked difference in the primary site [39 vs. 36], lymph nodes [221 vs. 208], pleural metastases [14 vs. 11] and brain metastasis [42 vs. 25]. Discordant finding in the primary tumor site was observed in 10 patients between [^68^Ga]Ga-DOTA.SA.FAPi and [^18^F]F-FDG scans with superior findings on the FAPi scan (Table 2). Five false-negative findings were observed on [^68^Ga]Ga-DOTA.SA.FAPi and eight on [^18^F]F-FDG.

**Figure 1:**
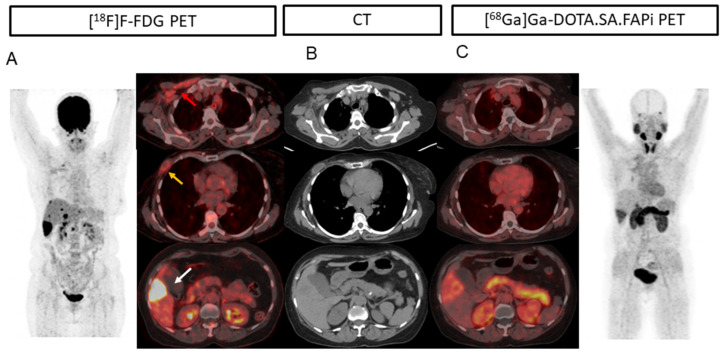
A 56-year-old patient with invasive ductal carcinoma (ER-/PR-/HER2+) underwent neoadjuvant chemotherapy, modified radical mastectomy, capecitabine, and lapatinib chemotherapy. On imaging, [^18^F]F-FDG PET/CT [(**A**), red arrow] showed uptake in the pectoralis major and minor muscle, which was not observed on [^68^Ga]Ga-DOTA.SA.FAPi PET/CT (**C**), suggesting inflammation. (**A**) well-defined cystic lesion with peripheral FDG uptake is noted in the right anterior chest wall at the post-operative site (Right modified radical mastectomy) (yellow arrow) but no uptake was observed on the FAPi scan (**B**). Compared to [^68^Ga]Ga-DOTA.SA.FAPi PET/CT (**B**), [^18^F]FDG PET/CT [(**A**), white arrow] showed intense uptake in the hypodense liver lesion (**B**) measuring 4.1 × 3 cm in segment VI, subcapsular location. In this patient FAPi could detect all the lesions corresponding to CT, but [^18^F]F-FDG detected false positive findings in the primary site and increased muscle uptake.

**Figure 2:**
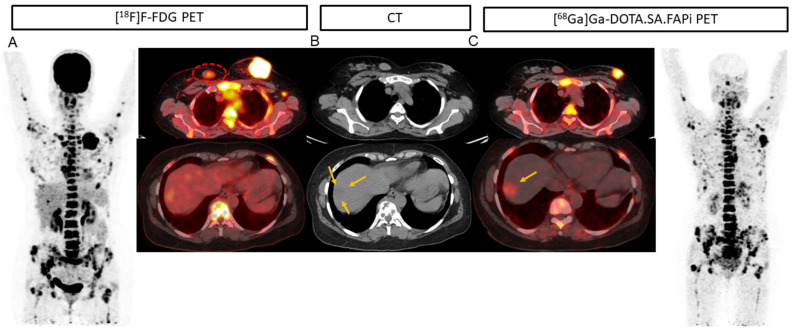
[^18^F]F-FDG PET/CT (**A**) and [^68^Ga]Ga-DOTA.SA.FAPi PET/CT (**B**) avid soft tissue density mass measuring 5.2 × 4.3 cm noted in the upper quadrant extending into the retro-areolar region and upper quadrants involving nipple-areola complex and overlying skin. A small well defined rounded soft tissue density lesion measuring 2 × 3 cm was noted in the upper inner quadrant of the right breast with mild FDG uptake-likely fibroadenoma (**A**, red dotted circle) but no uptake on [^68^Ga]Ga-DOTA.SA.FAPi scan. Due to increased physiological hepatic FDG uptake, the liver lesion (**A**) is masked in the^18^F-FDG PET/CT as compared to the [^68^Ga]Ga-DOTA.SA.FAPi PET/CT [(**C**), yellow arrow] and corresponding lesion on CT [(**B**), yellow arrows].

In one patient (Figure 1) with invasive ductal carcinoma (ER-/PR-/HER2+), on imaging, [^18^F]F-FDG PET/CT (A; red arrow) showed uptake in the pectoralis major and minor muscle suggesting inflammation. The same was not observed on [^68^Ga]Ga-DOTA.SA.FAPi PET/CT (B). A well-defined cystic lesion with peripheral FDG uptake is noted in the right anterior chest wall at the post-operative site (right modified radical mastectomy) (yellow arrow) but no uptake was observed on [^68^Ga]Ga-DOTA.SA.FAPi PET scan (B). In this patient FAPi could detect all the lesions corresponding to CT, but [^18^F]F-FDG detected false positive findings in the primary site and increased muscle uptake. Another example of FDG uptake in fibroadenoma was [^68^Ga]Ga-DOTA.SA.FAPi negative is depicted in Figure 2.

**Figure 3:**
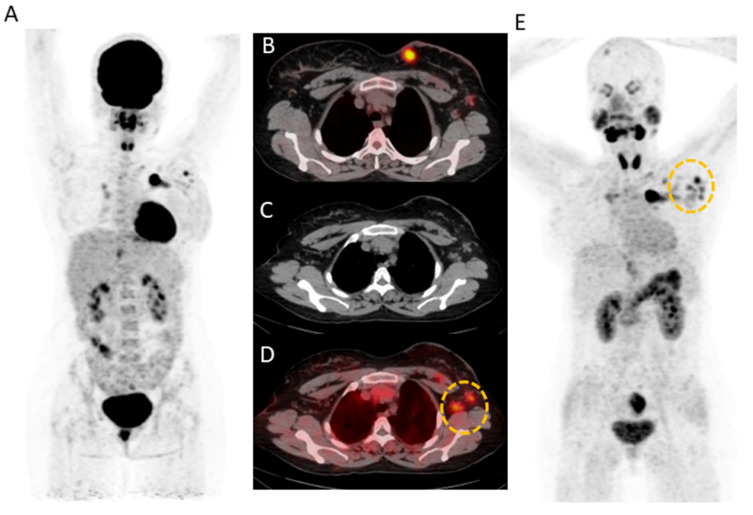
(**A**) 31-year-old female underwent modified radical mastectomy (ER+/PR+/Her2-) and 2 cycles of docetaxel chemotherapy. [^18^F]F-FDG (**A**,**B**) and [^68^Ga]Ga-DOTA.SA.FAPi (**E**) avid irregular mass (measuring 2.6 × 2.4 cm) noted in the breast upper inner quadrant. [^68^Ga]Ga-DOTA.SA.FAPi showed increased radiotracer uptake noted in the left axillary level I and II lymph node (**D**) corresponding to the CT (**C**) as compared to that of [^18^F]F-FDG (yellow circle). [^18^F]F-FDG shows diffuse non-specific radiotracer uptake in the entire axial skeleton, which is likely reactive (**A**).

[^68^Ga]Ga-DOTA.SA.FAPi detected 221/248 lymph nodes (89.1%) compared to 208/248 (83.8%) lesions detected by [^18^F]F-FDG. A 31-year-old female underwent modified radical mastectomy (ER+/PR+/Her2-) followed by chemotherapy and was evaluated for response assessment. In this patient, [^68^Ga]Ga-DOTA.SA.FAPi showed increased radiotracer uptake noted in the left axillary level I and II lymph node corresponding to the CT compared but no avidity was observed on [^18^F]F-FDG (yellow circle) (FN). On the other hand, [^18^F]F-FDG showed diffuse non-specific radiotracer uptake in the entire axial skeleton, which is likely reactive (FP) (Figure 3).

**Figure 4:**
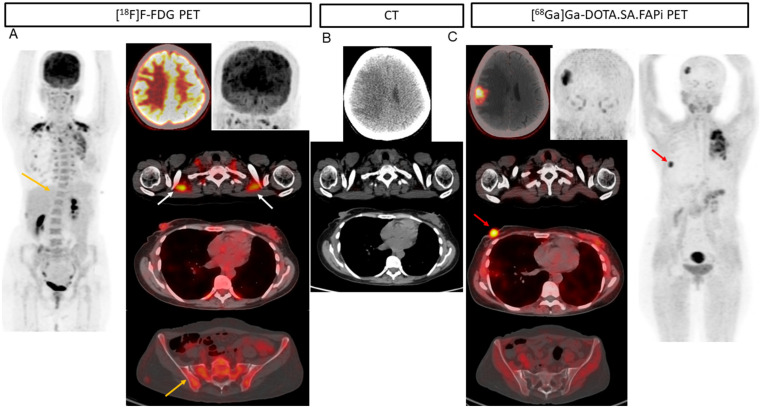
(**A**) 37-year-old patient with infiltrating ductal carcinoma underwent chemotherapy, Herceptin treatment, gamma knife for brain metastases, lapatinib, and capecitabine oral chemotherapy. A heterogeneous lesion measuring 1.7 × 1.4 cm was seen in the right parietal lobe associated with grade II perifocal vasogenic edema (**B**). (**A**) Due to the physiological uptake of FDG in the brain, lesions are not well demarcated (**A**). On [^68^Ga]Ga-DOTA.SA.FAPi PET scan, brain metastasis is appreciated in the MIP image itself (**C**). Brown fat uptake is seen on [^18^F]F-FDG ((**A**), white arrows), but no such findings were noted on [^68^Ga]Ga-DOTA.SA.FAPi PET scan (**C**). High-intensity uptake on [^68^Ga]Ga-DOTA.SA.FAPi in the right areola region corresponds to the mass in CT scan (**B**), but minimal radiotracer uptake on [^18^F]F-FDG PET scan (**A**). Diffuse non-specific bone marrow uptake was appreciated on [^18^F]F-FDG MIP (**A**) and transverse section ((**A**), yellow arrow) in the axial skeleton that was negative on CT (**A**,**C**) and [^68^Ga]Ga-DOTA.SA.FAPi (**C**) PET scans. [^68^Ga]Ga-DOTA.SA.FAPi clearly detected both primary and brain lesions on MIP itself (**C**).

**Figure 5:**
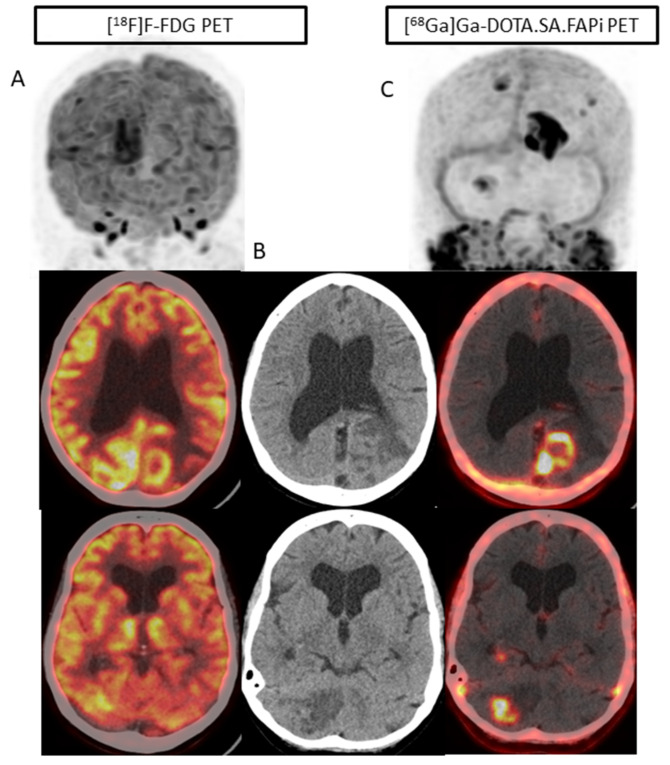
In a 54-year-old patient with bilateral carcinoma breast, the post lumpectomy of the right breast tumor and left radical mastectomy the histopathology revealed invasive ductal carcinoma (ER-/PR and HER2+). Multiple isodense lesions were noted in the right parietal and left occipital lobes (**B**), with surrounding edema. [^18^F]F-FDG PET/CT (**A**) underestimated the number of lesions as compared to [^68^Ga]Ga-DOTA.SA.FAPi PET/CT (**C**), as only one lesion was visualized on [^18^F]F-FDG PET/CT brain MIP images.

Among the 17 patients with liver metastases [^68^Ga]Ga-DOTA.SA.FAPi and CT accurately detected all 88 lesions (100%), but the accuracy decreased to 96.5% (85/88). The superiority of [^68^Ga]Ga-DOTA.SA.FAPi over [^18^F]F-FDG is portrayed in Figure 2. Due to increased physiological hepatic FDG uptake, the liver lesion (A) is masked in the [^18^F]F-FDG PET/CT as compared to the [^68^Ga]Ga-DOTA.SA.FAPi PET/CT (minimal FAPi physiological hepatic uptake) (C) and corresponding lesion on CT (B). [^68^Ga]Ga-DOTA.SA.FAPi had higher detectability of pleural metastases than [^18^F]F-FDG (15 vs. 13). [^68^Ga]Ga-DOTA.SA.FAPi definitely changed the imaging technique for brain metastases wherein [^68^Ga]Ga-DOTA.SA.FAPi accurately detected all 42 brain metastases, whereas a drastically low number of 25 were detected on [^18^F]F-FDG (sensitivity 100% vs. 59.5%, *p* < 0.0001) (Table 1) (Figure 4 and Figure 5).

### 2.4. Comparison of Uptake and TBRs in Tumor Lesions

The mean SULpeak and average values of lymph node metastases, liver metastases, and pleural metastases were significantly higher on [^68^Ga]Ga-DOTA.SA.FAPi than [^18^F]F-FDG imaging (*p* < 0.05). However, SULpeak values of the primary breast tumors, lung metastases, brain metastases, and bone metastases did not exhibit a statistically significant difference between the two imaging techniques (*p* > 0.05) (Table 1).

Interestingly, there was no significant difference in the SULpeak and average values in brain metastases, but remarkably high TBR values were observed on [^68^Ga]Ga-DOTA.SA.FAPi compared to that of [^18^F]F-FDG PET. [^68^Ga]Ga-DOTA.SA.FAPi imaging showed 10 brain metastases in one patient, while [^18^F]F-FDG showed only four lesions due to high background activity (Figure 1).

In addition to brain metastases, [^68^Ga]Ga-DOTA.SA.FAPi imaging yielded significantly higher TBRs in breast lesions as well as hepatic, bone, and lung metastases compared to [^18^F]F-FDG (*p* < 0.05) (Table 1). The median TBR ratio was favorable for [^68^Ga]Ga-DOTA.SA.FAPi over [^18^F]F-FDG in the primary and all metastatic lesions but statistically in case of lymph node, pleural metastases, liver and brain metastases (*p* < 0.05).

## 3. Discussion

Recent work has highlighted that FAP expression is markedly upregulated in various cancers and has gained interest as a potential small molecule inhibitor [9,10,11,12]. Cancer-associated fibroblasts have a direct involvement in the progression of metastatic breast carcinoma with decreased sensitivity to chemotherapy and hormonal therapy [13,14,15,16,17,18,19].

This retrospective study was conducted to compare the diagnostic value of [^18^F]F-FDG and [^68^Ga]Ga-DOTA.SA.FAPi PET/CT in breast cancer patients. According to our results, the patient-based analysis showed no significant difference between modalities, as opposed to the lesion-based analysis which showed [^68^Ga]Ga-DOTA.SA.FAPi superior to [^18^F]F-FDG PET/CT. Overall results suggest that [^68^Ga]Ga-DOTA.SA.FAPi detected more lesions and a higher tumor-background ratio compared to that of [^18^F]F-FDG. [^68^Ga]Ga-DOTA.SA.FAPi PET/CT showed more accurate and sensitive visualization compared to [^18^F]F-FDG PET/CT, especially in the primary tumor, lymph nodes, pleural, and brain metastases because of the good tumor delineation and high TBRs. These findings may help us to understand the superior lesion detection with [^68^Ga]Ga-DOTA.SA.FAPi has important implications for accurate management and developing future small molecule FAP inhibitor theranostic probes.

Tchou et al. [20] evaluated the expression of FAP in a panel of 52 human breast tumor samples using a combination of immunohistochemistry analyses. They observed an abundant expression of FAP in >70% of tumor samples even without a significant correlation with clinicopathologic factors. Tchou et al. [20] additionally investigated five fresh human breast tumor specimens and demonstrated that some of these FAP+ CD45+ cells were CD11b + CD14 + MHC-II+ indicating that they were likely tumor-associated macrophages (TAMs). Significant observation emerged that human breast TAMs also expressed FAP. These results strengthened the fact that future FAP-directed therapy may have dual therapeutic benefits targeting both stromal mesenchymal cells and immune cells such as TAMs. On the contrary, interestingly, a study by Ariga et al. [21] suggested that FAP expression in breast cancer was inversely correlated with breast cancer prognosis.

Similar to Komek et al. [4], in our study [^68^Ga]Ga-DOTA.SA.FAPi detected more primary tumor lesions, and higher TBR than [^18^F]F-FDG, but unlike Komek et al. [4], there was no difference in the uptake values. In one of our cases, avid FDG uptake was observed in a patient with fibroadenoma in the right breast but did not show FAP expression. Another patient developed post-operative seroma development at the surgical site of the right breast region. While [^18^F]F-FDG showed moderate avidity, the corresponding [^68^Ga]Ga-DOTA.SA.The FAPi scan was negative.

Furthermore, various lymph node involvements were correctly detected and showed complete concordance with CT in all the lesions by [^68^Ga]Ga-DOTA.SA.FAPi PET/CT in 36 out of 41 (87.8%) patients (five showed a mixed pattern of FP, FN, TP, and TN). A drastically low number of 26 out of 41 (63.4%) patients showed a complete concordance of FDG uptake in the lymph nodes corresponding to the CT morphology (15 patients showed mixed FDG uptake in the LNs).

Over 4.2% of the [^68^Ga]Ga-DOTA.SA.FAPi and 10.6% [^18^F]F-FDG scans reported mixed uptake in lung metastases. The size of metastatic foci yields low activity and false negative results with radiotracers, particularly in lymph node and lung metastases. In such circumstances, morphological characteristics on diagnostic CT may aid to distinguish malignant etiology from benign.

Regarding the bone metastases, due to the high disease burden, it was impractical to compare each lesion; however, the visual analysis did not reveal any difference in the detection rate between the two scans except for two patients who had diffuse non-specific uptake on [^18^F]F-FDG scan. Low activity uptake in the bone is an inflammatory sequel and the findings are consistent with findings from other studies in various cancers. [^68^Ga]Ga-DOTA.SA.FAPi demonstrated higher SUV values and TBR due to lower [^68^Ga]Ga-DOTA.SA.FAPi uptake in the normal bone tissue. Komek et al. [4] found a greater number of bone lesions with higher TBR on [^68^Ga]Ga-DOTA.SA.FAPi compared to [^18^F]F-FDG, but the difference was not significant.

[^68^Ga]Ga-DOTA.SA.FAPi outplayed [^18^F]F-FDG in terms of the number of liver lesions and TBR. In line with our findings, the majority of the studies have reported lower physiological uptake in the liver parenchyma with [^68^Ga]Ga-DOTA.SA.FAPi than [^18^F]F-FDG [4,5]. A similar scenario is a case of a 51-year-old breast cancer patient with liver metastases. [^18^F]F-FDG PET/CT showed minimal uptake (SULpeak: 2.2) in the hepatic lesions with a significantly higher uptake on [^68^Ga]Ga-DOTA.SA.FAPi (SULpeak: 9.9).

The superiority of [^68^Ga]Ga-DOTA.SA.FAPi is straightforward with a clear advantage over [^18^F]F-FDG PET/CT scans in the detection of brain metastases. Although this pattern is expected because of the FDG distribution in the brain, it represents a unique field for FAPi.

### 3.1. Limitations

Our study had some limitations. CECT scans were not acquired in all patients. Gold standard histopathology validation was performed in only the locoregional tumor lesions for all patients as it was impractical to assess biopsy from each lesion.

### 3.2. Future Prospects

More work in a larger patient population is needed to dissect the role of FAP in various histopathologic variants and hormone-receptor status within the tumor microenvironment and explore its role as a potentially targetable molecule in breast cancer treatment. The fundamental impact of comparing [^68^Ga]Ga-DOTA.SA.FAPi with [^18^F]F-FDG is the fact that different from FDG there is a clear theranostic option with FAP inhibitors. Based on [^68^Ga]Ga-DOTA.SA.FAPi there is a straightforward direction to subsequent therapies utilizing [^177^Lu]Lu.DOTAGA(SA.FAPi)_2_, representing the same targeting vector for the same oncological target.

## 4. Materials and Methods

This retrospective study was approved by the institute ethics committee of All India Institute of Medical Sciences, New Delhi, India. Patients were enrolled between April 2020 and August 2022. The study was conducted in the Department of Nuclear Medicine in collaboration with the Department of Medical Oncology at All India Institute of Medical Sciences and the Department of Chemistry, Johannes Gutenberg University, Mainz, Germany, which provided the DOTA.SA.FAPi molecule. Radiolabeling of [^68^Ga]Ga-DOTA.SA.FAPi was conducted as detailed in our previous publications [9,10,11,12]. In short, a mixture of 10 nmol of DOTA.SA.FAPi precursor, 1 mL ammonium acetate buffer pH 4, and [^68^Ga]Ga-chloride solution were heated at 95 °C for 10 min. After heating, the radiolabeled product was eluted through a Sep-Pak C18 Plus Light cartridge with 50% ethanol followed by 10 mL of normal saline. Quality control with sodium citrate buffer pH4 yielded > 95% radiochemical purity.

### 4.1. Patient Selection

Breast cancer patients with locally advanced or metastatic stage disease who underwent both [^68^Ga]Ga-DOTA.SA.FAPi PET/CT and [^18^F]F-FDG PET/CT scans within a 15-day time frame were included in the retrospective study. The inclusion criteria of the patients were; (i) patients > 18 years of age, (ii) breast cancer confirmed by histopathological evaluation, and (iii) patients who did not receive radiotherapy or chemotherapy within six weeks. Patients with a known inflammatory condition, dual malignancies, pregnant patients, and those unwilling to undergo two PET/CT scans were excluded from the study. Histopathological (HPE) evaluation was performed in all patients from the primary and suspicious lymph nodes. Immunohistochemistry analysis included evaluation of estrogen receptor (ER), progesterone receptor (PR), Ki-67, and HER2/neu. In patients with additional lesions on any scan, the HPE was evaluated in the corresponding lesion.

### 4.2. PET/CT Acquisition

According to the eligibility criteria, 47 patients were included in the study. Patients fasted at least 6 h before the [^18^F]F-FDG injection, but no such preparation was required for [^68^Ga]Ga-DOTA.SA.FAPi PET/CT scans. A normal blood glucose level in the peripheral blood was ensured before [^18^F]F-FDG PET/CT evaluation. Mean activities of 222 MBq [^18^F]F-FDG and 200 MBq [^68^Ga]Ga-DOTA.SA.FAPi were injected. Scans were acquired on a 128-slice GE Discovery 710 × 128 Slice PET/CT Scanner with a 40-mm detector at a 0.35-s rotation speed after 60 min of intravenous injection of both radiotracers. For all acquisitions, the patient was positioned in a supine position. The scans involved an initial scout image to define the field of view, followed by a CT and a PET scan. The CT scan involved a diagnostic dose CT with 300 to 380 mAs, 120 kVp, slice thickness 3.75 mm, and pitch 0.6. Spot views were acquired if required in brain metastases patients with a slice thickness of 1.25 mm on CT at 120 kVp, 300–380 mAs, and a pitch of 0.6. Images were processed and analyzed on a GE Xeleris workstation. Image acquisition and analysis for [^18^F]F-FDG and [^68^Ga]Ga-DOTA.SA.FAPi PET/CT involved a qualitative and quantitative comparison between the traces.

### 4.3. Data Interpretation

To assess the diagnostic ability of [^68^Ga]Ga-DOTA.SA.FAPi, a patient-based analysis was conducted in both primary and all metastatic lesions. Lesion-based analysis was feasible in the primary, lymph node, liver metastases, and brain metastases.

[^18^F]F-FDG and [^68^Ga]Ga-DOTA.SA.FAPi PET/CT scans were independently reviewed and processed by two nuclear medicine physicians with more than 15 years of experience in interpreting PET/CT data. They were blinded to the clinical history and HPE status of the patients. Any differences in the interpretation were discussed and settled with mutual agreement.

### 4.4. Data Analysis and Processing

The qualitative analysis involved visual judgment of radiotracer uptake which was validated by the morphological findings on diagnostic CT which was considered as the reference standard. For quantitative comparisons between the radiotracers, a 3D auto-contour ROI at a 40% threshold of SULpeak was carefully drawn around the site of [^18^F]F-FDG respective [^68^Ga]Ga-DOTA.SA.FAPi expressing lesions on transaxial images. The ROIs were presented as standardized uptake value (SUV) corrected for lean body mass: SULpeak and SULavg to quantitatively compare the uptake in the lesions between the radiotracers. The SUV values (peak, average, median, and range) were recorded for each site for both [^18^F]F-FDG and [^68^Ga]Ga-DOTA.SA.FAPi. The uptakes in the lesions on both scans were compared with the morphological features/characteristic on the CT counterpart. Tumor-to-background ratio (TBR) was calculated by dividing the SULpeak of the primary tumor/metastases with corresponding background SULpeak values.

### 4.5. Definitions

- True-positive (TP) lesion: active uptake in the lesion seen on [^18^F]F-FDG/[^68^Ga]Ga-DOTA.SA.FAPi PET/CT images and found to be positive on diagnostic CT/histological examination.
- False-positive (FP) lesion: active uptake in the lesion seen on [^18^F]F-FDG/[^68^Ga]Ga-DOTA.SA.FAPi PET/CT images and found to be negative on diagnostic CT/histological examination/clinical or radiological follow-up.
- True-negative (TN) lesion: No uptake seen on [^18^F]F-FDG/[^68^Ga]Ga-DOTA.SA.FAPi PET/CT images and the results on diagnostic CT/histological examination/clinical or radiological follow-up were also negative.
- False-negative (FN) lesion: a lesion that was missed in [^18^F]F-FDG/[^68^Ga]Ga-DOTA.SA.FAPi PET/CT images were found to be positive for malignancy at diagnostic CT/histological examination/clinical or radiological follow-up.

### 4.6. Statistical Analysis

Continuous variables were presented in terms of mean, median, standard deviation (SD), range, and interquartile range (IQR). [^18^F]F-FDG and [^68^Ga]Ga-DOTA.SA.FAPi uptakes were compared using paired Student’s *t*-test or Wilcoxon signed-rank test. The sensitivity, specificity, and accuracy of [^18^F]F-FDG and [^68^Ga]Ga-DOTA.SA.FAPi PET/CT examinations were calculated and compared. Statistical difference in the detection rate of primary tumors, lymph nodes, and visceral metastases between [^18^F]F-FDG and [^68^Ga]Ga-DOTA.SA.FAPi scans were analyzed by the McNemar test. Statistical analysis was performed using MedCalc statistical software (v15.0).

## 5. Conclusions

In conclusion, [^68^Ga]Ga-DOTA.SA.FAPi PET/CT enabled superior tracer uptake in primary tumors and metastatic lesions compared to [^18^F]F-FDG PET/CT. [^68^Ga]Ga-DOTA.SA.FAPi identified more abnormal lesions than [^18^F]F-FDG in all the primary and metastatic sites with a marked superiority in the primary tumor, lymph nodes, and brain metastases. The high FAP expression and remarkable TBR facilitate better detection efficiency of [^68^Ga]Ga-DOTA.SA.FAPi PET/CT and targeted systemic radionuclide therapy with [^177^Lu]Lu-DOTAGA(SA.FAPi)_2_ for various FAPi-expressing cancers. Unlike [^18^F]F-FDG imaging [^68^Ga]Ga-DOTA.SA.FAPi imaging is independent of blood glucose levels which could increase the ease of the procedure and diagnostic performance. It is also a cost-effective option in sites without a cyclotron facility.

## Figures and Tables

**Table 1:** Comparison of various parameters between [^18^F]F-FDG and [^68^Ga]Ga-DOTA.SA.FAPi PET/CT according to the lesion location.

Parameters	Imaging Method	Primary	Lymph Node Metastasis	Lung Metastasis	Pleural Metastases	Liver Metastases	Bone Metastases	Brain Metastases
**Patient-based analysis**								
	CT	30	41	26	8	17	25	15
	[^18^F]F-FDG	27 (90%)	38 (92.6%)	25 (96.1%)	6 (75%)	17 (100%)	24 (96%)	13 (86.6%)
	[^68^Ga]Ga-DOTA. SA.FAPi	29 (96.6%)	40 (97.5%)	25 (96.1%)	8 (100%)	17 (100%)	25 (100%)	15 (100%)
	*p*-value	0.3107	0.309	1.000	0.1432	1.000	0.865	0.748
**Lesion-based analysis**								
	CT	44	248	-	15	88	-	42
	[^18^F]F-FDG	36 (81.8%)	208 (83.8%)	-	11 (73%)	85 (96.6%)	-	25 (59.5%)
	[^68^Ga]Ga-DOTA. SA.FAPi	39 (88.6%)	221 (89.1%)	-	14 (93.3%)	88 (100%)	-	42 (100%)
	*p*-value	0.001	0.0001	-	0.096	0.785	-	0.0001
**SULpeak**								
	[^18^F]F-FDG	5.8 (2.5–11.7)	3.8 (2.4–6.6)	3.7 (2.3–9)	5.3 (2–8)	5.3 (3.1–11.9)	8.1 (4.5–11.6)	5.5 (3.5–6.6)
	[^68^Ga]Ga-DOTA. SA.FAPi	6.6 (4.1–11.9)	4.9 (2.9–7.8)	4.6 (3.2–7.2)	9.8 (3.2–9.2)	10.3 (6–4)	9.4 (5.4–15.4)	8.8 (7.6–10.7)
	*p*-value	0.3369	0.0453	0.523	0.0001	0.0419	0.114	0.1309
**SULavg**								
	[^18^F]F-FDG	3.8	2.1 (1.2–3.8)	3.7 (2.3–9)	2.6 (1.7–4.4)	3.9 (1.6–7.1)	4.7 (2.4–7.02)	3.2 (1.7–3.9)
	[^68^Ga]Ga-DOTA. SA.FAPi	5.4	2.8 (1.6–5.2)	4.6 (3.2–7.2)	5.8 (3–7)	5.5 (3.3–11.7)	5.3 (2.9–8.6)	5.3 (4.3–6.8)
	*p*-value	0.351	0.0431	0.523	0.0001	0.017	0.330	0.139
**TBR (SUVpeak)**								
	[^18^F]F-FDG	0.9 (0.1–3.4)	1.3 (0.4–3.6)	1.62 (1.1–4.08)	1.6 (0.6–2.3)	1.8 (1.2–3.2)	2.87 (1.96–0.13)	6.8 (1.9–9.8)
	[^68^Ga]Ga-DOTA. SA.FAPi	1.2 (0.2–4.3)	2.3 (0.9–4.2)	1.8 (1.1–2.9)	3.2 (1.1–4.2)	3.8 (2.7–6.6)	4.4 (3.2–9.3)	22.3 (11.6–35.6)
	*p*-value	0.5434	0.0129	0.3570	0.0023	0.0472	0.134	<0.0001

**Table 2:** Discordance in the detection of the primary tumor.

Patient S.No	CT	[^68^Ga]Ga-DOTA.SA.FAPi	[^18^F]F-FDG
11.	0	0	1
12.	1	1	0
18.	1	1	0
21.	2	1	1
24.	2	1	1
25.	0	0	1
29.	2	2	1
32.	4	2	2
35.	1	0	0
37.	0	0	1

## Data Availability

Data is contained within the article.
